# Tempering Proactive Cognitive Control by Transcranial Direct Current Stimulation of the Right (but Not the Left) Lateral Prefrontal Cortex

**DOI:** 10.3389/fnins.2017.00282

**Published:** 2017-05-23

**Authors:** Carlos J. Gómez-Ariza, María C. Martín, Julia Morales

**Affiliations:** ^1^Department of Psychology, University of JaénJaén, Spain; ^2^Department of Psychology, Loyola Andalucía UniversitySevilla, Spain

**Keywords:** dorsolateral prefrontal cortex, inferior frontal junction, executive control, cognitive flexibility, inhibition, tDCS

## Abstract

Behavioral and neuroimaging data support the distinction of two different modes of cognitive control: proactive, which involves the active and sustained maintenance of task-relevant information to bias behavior in accordance with internal goals; and reactive, which entails the detection and resolution of interference at the time it occurs. Both control modes may be flexibly deployed depending on a variety of conditions (i.e., age, brain alterations, motivational factors, prior experience). Critically, and in line with specific predictions derived from the dual mechanisms of control account (Braver, 2012), findings from neuroimaging studies indicate that the same lateral prefrontal regions (i.e., left dorsolateral cortex and right inferior frontal junction) may implement different control modes on the basis of temporal dynamics of activity, which would be modulated in response to external or internal conditions. In the present study, we aimed to explore whether transcraneal direct current stimulation over either the left dorsolateral prefrontal cortex or the right inferior frontal junction would differentially modulate performance on the AX-CPT, a well-validated task that provides sensitive and reliable behavioral indices of proactive/reactive control. The study comprised six conditions of real stimulation [3 (site: left dorsolateral, right dorsolateral and right inferior frontal junction) × 2 (polarity: anodal and cathodal)], and one sham condition. The reference electrode was always placed extracephalically. Performance on the AX-CPT was assessed through two blocks of trials. The first block took place while stimulation was being delivered, whereas the second block was administered after stimulation completion. The results indicate that both offline cathodal stimulation of the right dorsolateral prefrontal cortex and online anodal stimulation of the right inferior frontal junction led participants to be much less proactive, with such a dissociation suggesting that both prefrontal regions differentially contribute to the adjustment of cognitive control modes. tDCS of the left-DLPFC failed to modulate cognitive control. These results partially support the predictions derived from the dual mechanisms of control account.

## Introduction

A distinctive characteristic of human cognition is its flexibility. As human beings we have the ability to promptly adjust behavior to efficiently deal with changing internal and external conditions. While cognitive flexibility is thought to strongly rely on control mechanisms in charge of regulating and coordinating thoughts and actions in a goal-driven manner, extensive debate still exists on what precise neurocognitive mechanisms enable flexibility and how they are implemented in the brain (Rougier et al., 2005; Braver et al., 2009).

An influential theory on cognitive control is the dual mechanisms of control account (Braver et al., 2007; Braver, 2012). According to this theory, two different cognitive control modes, namely proactive and reactive, may be flexibly deployed to prompt goal-directed actions or thoughts and suppress inappropriate ones. Thus, the proactive control mode is proposed to act by actively maintaining task-relevant information in a sustained manner to bias behavior in accordance with internal goals. From this perspective, proactive control may be understood in terms of early selection to prevent interference from cognitively demanding events (Miller and Cohen, 2001; Braver, 2012). Conversely, the reactive control mode is thought to act by detecting and resolving interference at the time it occurs and may be conceptualized as a late corrective function to deal with already arisen conflicts (Braver et al., 2009).

A remarkable characteristic of the dual mechanisms of control theory lies in the assumption that proactive and reactive control may be dissociated depending on the dynamic and location of brain activity (Braver et al., 2009; Braver, 2012). Thus, proactive control is expected to be mainly associated with anticipatory and sustained activation of the lateral prefrontal cortex (PFC), which would reflect goal-maintenance activity, whereas reactive control would entail transient activity of the lateral PFC (in addition to activity in other brain regions), which would reflect detection and/or resolution of interference only at specific times. Hence, the dual mechanisms of control account postulates that the same lateral PFC regions may implement different cognitive control modes on the basis of temporal dynamics of activity, which would be modulated in response to external or internal conditions (Braver, 2012).

A wealth of data support the distinction between proactive and reactive control and reveal that between-groups as well as intra-individual differences exist in the deployment of such cognitive control modes (e.g., Paxton et al., 2006, 2008; Locke and Braver, 2008; Chatham et al., 2009; Braver et al., 2009; Lesh et al., 2013; Morales et al., 2013, 2015). Thus, for example, it has been shown that healthy young adults exhibit behavioral performance and brain activity (i.e., sustained lateral PFC activation) that are consistent with a predominantly proactive control style, whereas healthy older adults are usually more reactive presumably because proactive control is cognitively more demanding (e.g., Braver et al., 2001; Paxton et al., 2008). In a similar vein, people with altered PFC functions (i.e., individuals with schizophrenia) exhibit behavioral performance (e.g., Barch et al., 2001; Holmes et al., 2005; Yoon et al., 2008) and reduced anticipatory activation in the lateral PFC (e.g., MacDonald and Carter, 2003) consistent with impoverished proactive cognitive control.

Of particular relevance to the current study, there is evidence that some experimental manipulations and everyday life experiences may impact on which and how cognitive control modes are deployed. Thus, task-strategy training has shown to be effective at making older adults and people with schizophrenia more able to engage in proactive, healthy young adult-like cognitive control (Paxton et al., 2006; Braver et al., 2009; Edwards et al., 2010). Conversely, young adults exhibit a shift from proactive to reactive control under conditions of reward-based monetary incentives (Locke and Braver, 2008). More recently, and on the basis of behavioral and brain activity data, it has been argued that early bilinguals are able to selectively adjust proactive and reactive control more efficiently than their monolingual peers (Morales et al., 2013, 2015), with such an ability being related to the bilinguals' extensive practice in coordinating two languages in their minds (for a discussion of this issue, see Kroll and Bialystok, 2013).

Altogether, the above-mentioned findings support the distinction of at least two modes of cognitive control that may be flexibly used depending on a variety of conditions (i.e., age, brain alterations, motivational factors, prior experience). In addition, and in accordance with specific predictions derived from the dual mechanisms of control account (Braver, 2012), some of these findings suggest that certain regions within the lateral PFC are involved in both proactive and reactive control, although with different temporal dynamics of activity. Braver et al. (2009), by using functional magnetic resonance imaging (fMRI), reported that the left dorsolateral PFC (left DLPFC; BA 9/46) and the right inferior frontal junction (right IFJ; BA 44/6) show shifts in activation dynamics that are associated with shifts, at the behavioral level, in the mode of cognitive control being deployed. Thus, for example, it has been shown that the left DLPFC and the right IFJ increase their sustained activity in older adults after being trained in proactive control, while both regions shift to a more transient pattern of activity in younger adults when they are motivated to be more reactive by means of monetary incentives (Braver et al., 2009). Hence, it would appear that certain experimental manipulations known to shift the default mode of cognitive control (as reflected in behavioral measures) also impact differentially on the activity in the lateral PFC. Importantly, while these data are suggestive of the role that specific prefrontal areas may play in the weighting of proactive and reactive control, such evidence is correlative given that neuroimaging research is unable to provide causal links between brain areas and task performance.

With the idea of exploring a more direct relationship between the lateral PFC and the deployment of proactive and reactive control, in the present study we use transcranial direct current stimulation (tDCS) as a means of modulating cortical excitability (e.g., Antal et al., 2004; Romero Lauro et al., 2014). As found to be the case with other non-invasive brain stimulation techniques, it is thought that tDCS may be beneficial to better understand the neural substrates of behavior (Berryhill et al., 2014; Filmer et al., 2014; Bestmann et al., 2015).

tDCS involves the delivery of a constant weak current (usually 1–2 mA) which is typically applied over the cerebral cortex through two surface electrodes. At least one of the electrodes is placed on the participant's scalp (over the region of interest), while the other is positioned over a location of lesser interest (either on the scalp or extracephalically on the contralateral shoulder/arm). The electrical current flows from one electrode (anode) to the other (cathode) over a period of time (5–20 min), which is thought to modulate cortical excitability in the stimulated region as well as in anatomically connected regions (Romero Lauro et al., 2014). At a cellular level, anodal tDCS leads to increased neuronal excitability (depolarization) in the area under the electrode, whereas cathodal tDCS produces the opposite effect (via hyperpolarization), with possible post-stimulation effects being associated with long-term potentiation-like mechanisms (Liebetanz et al., 2002). Importantly, while the precise behavioral effects of stimulating prefrontal areas are still difficult to predict (Jacobson et al., 2012), tDCS is considered to be a valuable technique to gain understanding of the involvement of certain brain areas (or networks) in a specific cognitive function (Filmer et al., 2014; Bestmann et al., 2015; Fertonani and Miniussi, 2016).

The specific aim of the present study was to explore whether anodal/cathodal tDCS over either the left DLPFC or the right IFJ would differentially affect performance on the AX-CPT, a well-validated task that has shown to provide sensitive and reliable behavioral indices of proactive/reactive control (i.e., Braver et al., 2009; Chiew and Braver, 2013). Because of the demonstrated non-linearity of the induced effects (anodal tDCS does not necessarily produce enhanced performance nor does cathodal tDCS always lead to impairments in performance; see Fertonani and Miniussi, 2016), we were agnostic about the selectivity of the putative effects of tDCS over our regions of interests (see Bestmann et al., 2015 for arguments in support of this approach). However, to the extent that these regions (in their own right or as part of a wider network) play a role in proactive/reactive control, one may predict tDCS (anodal and/or cathodal) to change performance relative to sham. Hence, and on the basis of previous fMRI studies using the AX-CPT, we hypothesized that tDCS over both regions within the lateral prefrontal cortex (left DLPFC and right IFJ) would modulate the type of cognitive control strategy deployed by young adults who, as mentioned above, tend to exhibit a proactive control style. Specifically, we employed a modified version of the AX-CPT used by Morales et al. (2013; see also Ophir et al., 2009), whereby participants are presented with cue-probe pairs and are told to respond “yes” to a target X-probe when preceded by an A-cue and to respond “no” to any other cue-target combinations. Target trials (AX) occur throughout the experiment with very high frequency (70%) and different types of non-target trials are intermixed with them. In 10% of the trials, a non-cue letter precedes the target (BX); in another 10% a distractor follows the cue (AY); and in the remaining 10% of the trials a non-cue letter precedes a distractor (BY).

**Figure 1:**
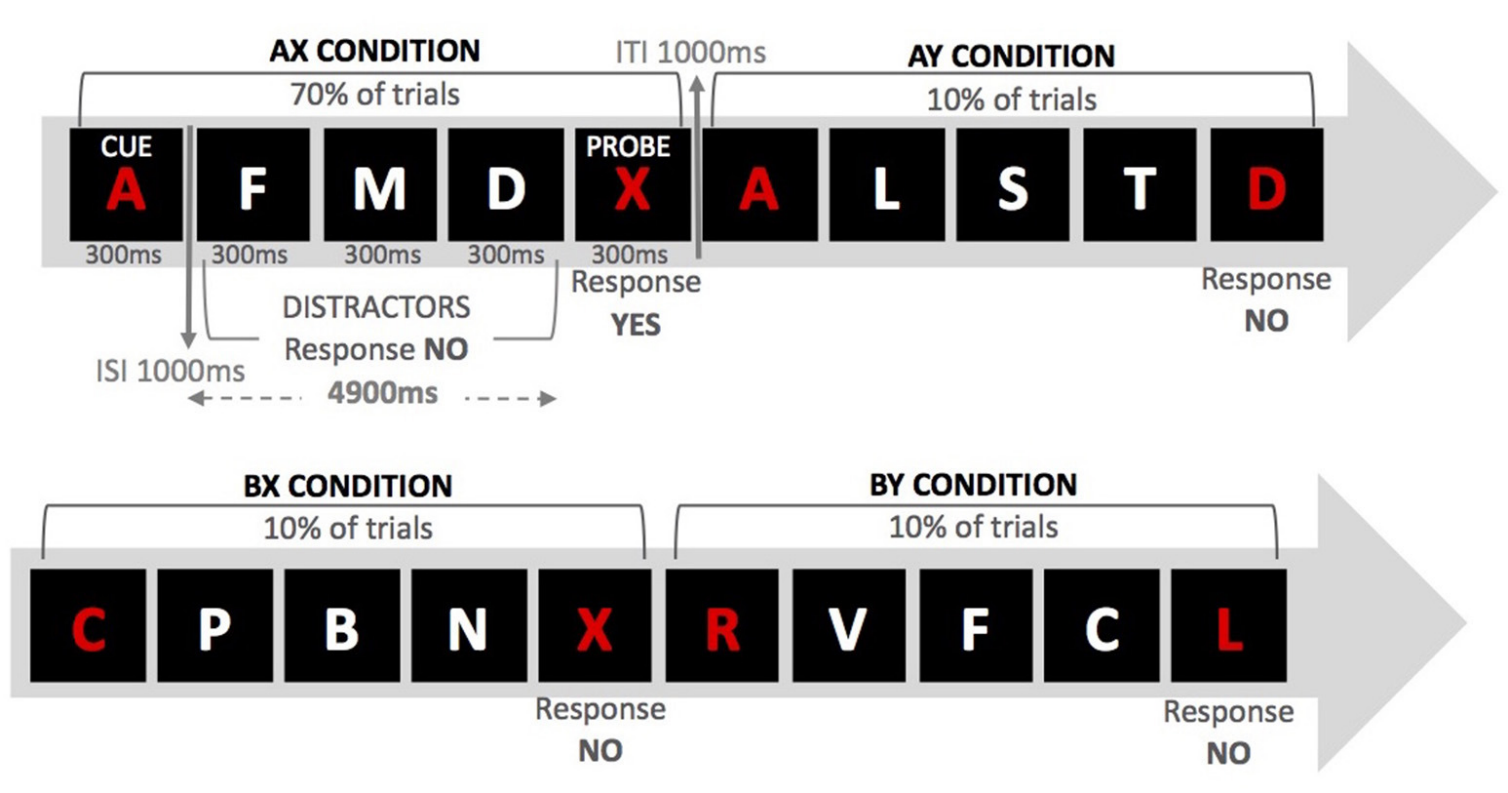
**Examples of series of events for the four trial types in the AX-CPT**.

In the AX-CPT, proactive and reactive control is usually assessed on the basis of participants' performance on conflict (AY and BX) trials (see Figure 1). High reliance on cue processing, which requires goal maintenance in accordance with the information provided by the cue, would produce high target expectancies when the A cue is presented (note that A-cues signal X probes for 70% of the trials). Hence, enhanced proactive control is expected to increase AY errors and reduce BX errors (since the cue in BX trials does not signal a “yes” response). On the contrary, high reliance on reactive control is expected to increase errors in BX trials (the probe signals a “yes” response) as well as decrease AY errors (the probe does not signal a “yes” response). Following previous work (i.e., Braver et al., 2009), in the present study we computed two different proactive indices (taken from error rates and RTs in AY and BX trials), which give a measure of the relative tendency toward proactive control (the higher the score, the higher the tendency). To the extent that the left DLPFC and the right IFJ are directly involved in the weighting of proactive and reactive control, one would expect tDCS over these regions to modulate the proactive index relative to the control (sham) condition. In other words, we aimed to explore whether the default cognitive control mode used by young participants would change as a result of applying tDCS over specific regions within the lateral prefrontal cortex.

**Figure 2:**
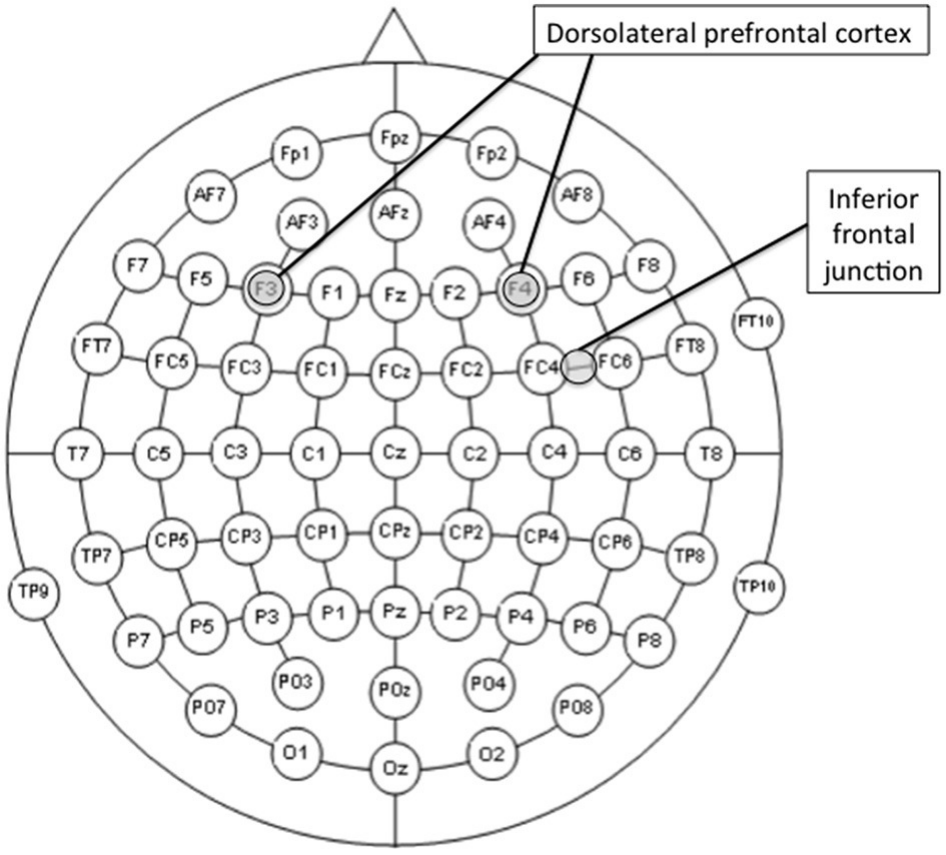
**Locations of the center of the electrodes of interest (F3, F4, or midway between FC4 and FC6 according to the 10-10 international system for EEG electrode placement) for the stimulation conditions**. The reference electrode was always placed over the contralateral shoulder.

The study comprised six conditions of real tDCS [3 (site; right DLPFC, left DLPFC, and right IFJ) × 2(polarity; anodal and cathodal)], and one fake (sham) stimulation to serve as the control condition. As previously described, the left DLPFC and the right IFJ were regions of interest because they have shown to be involved in both proactive-to-reactive and reactive-to proactive shifts. However, we also decided to stimulate the right DLPFC for this region to serve as a control site with which to compare the real tDCS over the two regions of main interest. The rationale behind this decision was twofold. On the one hand, and unlike its left counterpart, the right DLPFC has shown to be involved only in shifting from reactive to proactive control (see Braver et al., 2009), which might suggest either a more limited engagement of this region in the balance of both modes of cognitive control or a main involvement in proactive control. On the other hand, and due to the relative proximity between the right DLPFC and the right IFJ (see electrode montage in Figure 2), stimulating the former would provide us with a suitable condition to learn how site-specific the stimulation of the latter may be.

**Figure 3:**
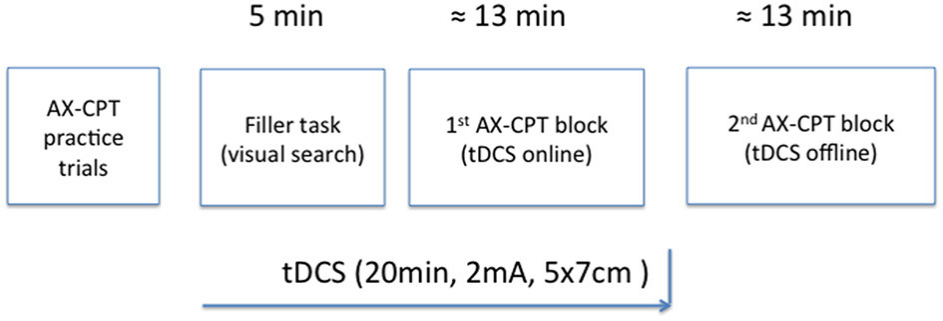
**Schematic representation of the experimental procedure**.

Finally, the effect of the timing of tDCS over performance on the AX-CPT was assessed through two blocks of experimental trials (see Figure 3). The first (online) block took place while tDCS was being delivered (or simulated in the case of the sham condition), while the second (offline) block was administered after a short break and only after stimulation completion. The rationale behind this procedure was, on one hand, to learn whether the potential effect of tDCS on cognitive control, as measured using the AX-CPT, persists beyond the stimulation period. This issue is not a trivial one, given that online and offline effects of tDCS are thought to be mediated by distinct neural mechanisms. Specifically, whereas online tDCS effects seem to be related to membrane polarization (e.g., Purpura and McMurtry, 1965; Stagg and Nitsche, 2011), long-term potentiation-like mechanisms seem to underlie offline tDCS-induced effects (e.g., Liebetanz et al., 2002; Fritsch et al., 2010; Stagg and Nitsche, 2011). On the other hand, since previous research has shown that engaging participants in a behavioral task concurrently with brain stimulation makes its aftereffects more specific (e.g., Feurra et al., 2013; Bortoletto et al., 2015), the present approach would allow us to learn how polarity (the only tDCS parameter manipulated here) and stimulation site modulate the brain activation induced by the AX-CPT. Though it is now recognized that task-induced activation interacts with the effect of stimulation non-linearly, further research on this question is required (Silvanto et al., 2008; Romei et al., 2016).

## Methods

### Participants

One hundred and seventy students from the University of Jaén volunteered to take part in the study in exchange for course credit (73% female: Age: *M* = 20.48, *SD* = 2.91). All participants were right-handed as assessed by the Edinburgh Inventory (Oldfield, 1971), and were randomly assigned to one of the seven experimental conditions. Based on previous studies that observed reliable between-groups differences with the AX-CPT using from 16 to 24 participants per group (i.e., Paxton et al., 2008; Edwards et al., 2010; Morales et al., 2013), we decided, prior to starting the experiment, to have 24 participants in each stimulation condition. Four participants were excluded from the study because of impedance problems (>5 kΩ as defined in the default mode of the stimulation device) and a further two participants were removed from the analyses because they exhibited extremely low performance (above 45% errors in the control trials). Thus, all the groups comprised 24 participants except anode left DLPFC, cathode left DLPFC, cathode right DLPFC, and sham, which comprised 23.

The study was approved and carried out in accordance with the recommendations of the Research Ethics Committee of the University of Jaén. All participants were provided with information about the study and gave written informed consent in accordance with the Declaration of Helsinki.

Participants were finally accepted to participate on the basis of their responses in a screening questionnaire that included the following exclusion criteria: history of neurological or psychiatric disorder, drug abuse, susceptibility to seizures, migraines, regular medication, implants, or neurosurgery. At the end of the experimental session, all participants completed a questionnaire on tDCS adverse effects (Brunoni et al., 2011). None of them reported major complaints or discomfort associated with stimulation.

### Materials and procedure

Participants were tested individually on the experimental task. Each session lasted approximately 60 min. We employed the modified version of the AX-CPT used by Morales et al. (2013) (see Figure 1). For this procedure, participants were presented with a sequence of letters for 300 ms each in the center of a black screen. The letters were displayed on a cue-probe basis so that 4,900 ms elapsed between presentation of cue and probe. The intertrial interval was 1,000 ms. Participants were instructed to maintain the cue in memory (either the letter “A” or any other letter except “X,” “K,” and “Y,” due to their perceptual similarity with “X”) until they saw the probe (either the letter “X” or any other letter except “A,” “K,” or “Y”). Whenever they saw the cue “A” followed by probe “X,” they were to respond by pressing the “yes” button. For any other possible cue-probe combination, participants were told to press the “no” button. Demands for goal maintenance were introduced by presenting three distractor letters between every cue-probe pair. The cues and the probes were red, whereas the distractors (any letter except “A,” “X,” and “Y”) were always white. Distractors were each presented for 300 ms with a 1,000 ms interval between letters. Participants were to respond with a “no” button press to the distractors.

Each session started with the practice block, followed by a distractor task and two experimental blocks (see Figure 2). The practice block was made up of 10 trials including all four possible experimental conditions: AX (an “A” cue followed by an “X” probe); BX (an “X” probe preceded by a non-A-cue); AY (any probe but “X” preceded by the letter “A”); and BY (any cue but “A” and any probe but “X”). Participants were provided with feedback on accuracy and RT after each practice trial. After completing the practice block, participants were instructed to complete a pen-and-paper visual search task (a distractor task, for a 5-min time interval) and then tDCS commenced. When the 5-min interval elapsed, participants were asked to stop performing the distractor task and start the first experimental block. After finishing the first block, participants were told to take a short break before starting the second block. This procedure aimed to ensure that the entire first block was performed with stimulation online and the second block without it (Nitsche and Paulus, 2000; Stagg and Nitsche, 2011). The interval between the two experimental blocks was no longer than 2 min. In both practice and experimental phases, AX trials occurred for 70% of the time, whereas each of the remaining experimental conditions appeared for 10% of the time.

### tDCS

Direct current was delivered through a battery-driven stimulator (neuroConn DC-STIMULATOR) and was applied through a pair of rubber electrodes (5 × 7 cm) covered with saline-soaked sponges. The electrode montage was based on the 10-10 international system for EEG electrode placement (Jurcak et al., 2007). The electrode of interest was placed, depending on the group, over the left DLPFC (F3), the right DLPFC (F4), or the right IFJ (FC6h: midway between FC4 and FC6; see Figure 3). The reference electrode was always placed extracephalically (contralateral shoulder) to minimize its effect on the brain (Noetscher et al., 2014). Stimulation lasted 20 min with a constant current of 2 mA (current density = 0.057 mA/cm^2^) and was faded in and out with an 8 s ramp. The same electrode montages were randomly used for sham stimulation, which lasted for 30 s.

## Results

Statistical analyses were performed on error rates and mean reaction times (see Table 1). Responses under 100 ms and over 1,000 ms (<3% of the data) were removed from the analyses. Performance was examined by means of analyses of variance (ANOVA) with group (stimulation condition: anodal right DLPFC, cathodal right DLPFC, anodal left DLPFC, cathodal left DLPFC, anodal right IFJ, cathodal right IFJ, and sham) as a between-participants factor, and block (tDCS online vs. tDCS offline) as a within-participant factor. First we report analyses for target (AX) and control (BY) trials, and then we describe performance for conflict (AY and BX) trials.

**Table 1:** **Mean error rates and reaction times (ms) for each trial type as a function of stimulation group and block (tDCS timing)**.

		**Block 1 (tDCS online)**				**Block 2 (tDCS offline)**			
		**AX**	**AY**	**BX**	**BY**	**AX**	**AY**	**BX**	**BY**
Sham	Errors RT	0.04 (0.04) 373 (41)	0.23 (0.18) 532 (69)	0.07 (10) 267 (39)	0.06 (0.08) 293 (58)	0.03 (0.04) 351 (45)	0.24 (0.20) 519 (64)	0.13 (0.17) 266 (71)	0.05 (0.09) 277 (61)
Anodal Left DLPFC	Errors RT	0.04 (0.04) 399 (61)	0.23 (0.13) 562 (107)	0.11 (0.11) 286 (58)	0.05 (0.08) 324 (92)	0.06 (0.06) 359 (61)	0.27 (0.18) 502 (102)	0.14 (0.16) 264 (55)	0.10 (0.11) 287 (75)
Cathodal Left DLPFC	Errors RT	0.07 (0.05) 378 (53)	0.32 (0.16) 527 (92)	0.14 (0.17) 280 (68)	0.10 (0.12) 311 (86)	0.07 (0.08) 356 (63)	0.34 (0.21) 503 (86)	0.16 (0.14) 243 (72)	0.10 (0.09) 277 (94)
Anodal Right DLPFC	Errors RT	0.07 (0.06) 374 (71)	0.24 (0.15) 529 (80)	0.11 (0.12) 290 (80)	0.06 (0.10) 295 (78)	0.07 (0.09) 358 (75)	0.27 (0.23) 507 (83)	0.14 (0.16) 257 (86)	0.08 (0.10) 282 (70)
Cathodal Right DLPFC	Errors RT	0.05 (0.04) 362 (50)	0.24 (0.17) 532 (87)	0.18 (0.21) 281 (84)	0.12 (0.08) 314 (84)	0.04 (0.06) 355 (49)	0.24 (0.20) 493 (101)	0.22 (0.19) 259 (56)	0.15 (0.14) 288 (107)
Anodal Right IFJ	Errors RT	0.04 (0.03) 373 (50)	0.21 (0.19) 524 (71)	0.15 (0.12) 264 (48)	0.08 (0.08) 296 (72)	0.07 (0.04) 346 (43)	0.30 (0.19) 499 (59)	0.15 (0.20) 236 (43)	0.12 (0.14) 271 (78)
Cathodal Right IFJ	Errors RT	0.04 (0.04) 388 (79)	0.25 (0.18) 541 (82)	0.11 (0.12) 304 (116)	0.05 (0.07) 312 (95)	0.05 (0.06) 376 (87)	0.33 (0.32) 526 (104)	0.20 (0.28) 276 (93)	0.13 (0.28) 306 (108)

*Standard deviations are presented in brackets (DLPFC, Dorsolateral Prefrontal Cortex; IFJ, Inferior Frontal Junction)*.

### Performance in target (AX) and control (BY) trials

The ANOVA group × block on errors in AX (target) trials only revealed a marginal effect of block, *F*_(1, 157)_ = 3.55, MSE = 0.00, *p* = 0.06, partial η^2^ = 0.02 [group: *F*_(6, 157)_ = 1.44, MSE = 0.01, *p* = 0.20, partial η^2^ = 0.05; interaction: *F* < 1, partial η^2^ = 0.04]. The participants' performance was somewhat worse during the second block of trials (online: *M* = 0.05, *SD* = 0.04; offline: *M* = 0.06, *SD* = 0.07).

Regarding errors in BY trials, the ANOVA showed the effect of block to be statistically significant, *F*_(1, 155)_ = 6.20, MSE = 0.01, *p* = 0.01, partial η^2^ = 0.04. Participants made more errors in the second (offline) block (*M* = 0.11, *SD* = 0.15) than they did in the first (online) block (*M* = 0.07, *SD* = 0.09). No other source of variability reached statistical significance [group: *F*_(6, 155)_ = 1.83, MSE = 0.02, *p* = 0.10, partial η^2^ = 0.07; interaction: *F* < 1, partial η^2^ = 0.03].

The same analyses were conducted on RTs for correct responses to AX and BY trials. The ANOVA on RTs in AX trials also failed to show an effect of group (*F* < 1, partial η^2^ = 0.02). Block was statistically significant, *F*_(1, 158)_ = 62.29, MSE = 576, *p* < 0.001, partial η^2^ = 0.28, with RTs being faster in the offline block (*M* = 358, *SD* = 58; online: *M* = 379, *SD* = 61). The interaction group × block reached statistical significance, *F*_(6, 158)_ = 2.43, MSE = 576, *p* = 0.02, partial η^2^ = 0.08. Bonferroni-corrected *post-hoc* comparisons revealed that the between-blocks differences in RTs were only reliable in the anodal left DLPFC and the anodal right IJF groups.

Finally, the ANOVA on RTs in BY trials showed that only the effect of block was statistically significant, *F*_(1, 155)_ = 13.18, MSE = 3,094, *p* < 0.001, partial η^2^ = 0.08 [group: *F* < 1, partial η^2^ = 0.02; interaction: *F* < 1, partial η^2^ = 0.02]. Participants responded to BY trials faster in the offline block (*M* = 290, *SD* = 92) than in the online block (*M* = 313, *SD* = 87).

### Performance in conflict (AY and BX) trials

First we performed a mixed ANOVA on the absolute error rates with group, block, and type of trial as factors. The analysis revealed statistically significant effects of trial type [*F*_(1, 157)_ = 82.99, MSE = 0.03, *p* < 0.001, partial η^2^ = 0.35; participants made more errors in AY (*M* = 0.26, *SD* = 0.17) than in BX trials (*M* = 0.15, *SD* = 0.14)] and block [*F*_(1, 157)_ = 10.15, MSE = 0.03, *p* < 0.001, partial η^2^ = 0.06; and more errors were made in block 2 (*M* = 0.22, *SD* = 0.18) than in block 1 (*M* = 0.18, *SD* = 0.13)]. No other source of variability reached statistical significance [all with *F* < 1 except trial x group: *F*_(6, 157)_ = 1.18, MSE = 0.03, *p* = 0.32, partial η^2^ = 0.04, and trial × block × group: *F*_(6, 157)_ = 1.12, MSE = 0.01, *p* = 0.35, partial η^2^ = 0.04].

The ANOVA on RTs showed the same pattern of statistical significance. There were main effects of trial type [*F*_(1, 150)_ = 1,835.11, MSE = 5,412, *p* < 0.001, partial η^2^ = 0.92; AY trials (*M* = 521, *SD* = 107) required more time than BX trials (*M* = 270, *SD* = 90)] and block [*F*_(1, 150)_ = 34.82, MSE = 3,095, *p* < 0.001, partial η^2^ = 0.19; participants responded faster to the second block (*M* = 382, *SD* = 94) than to the first block (*M* = 409, *SD* = 93)]. None of the interaction effects were shown to be reliable (all with *F* < 1).

#### Proactive indices

For each participant we computed a composite measure of proactive control on the basis of his/her performance in the two trials involving conflict resolution (AY and BX). Following previous work (e.g., Braver et al., 2009; Chiew and Braver, 2013), this measure was computed as (AY − BX)/(AY + BX) for errors and RTs, with a correction where errors were equal to zero [(error + 0.5)/(frequency of trials + 1)].

**Figure 4:**
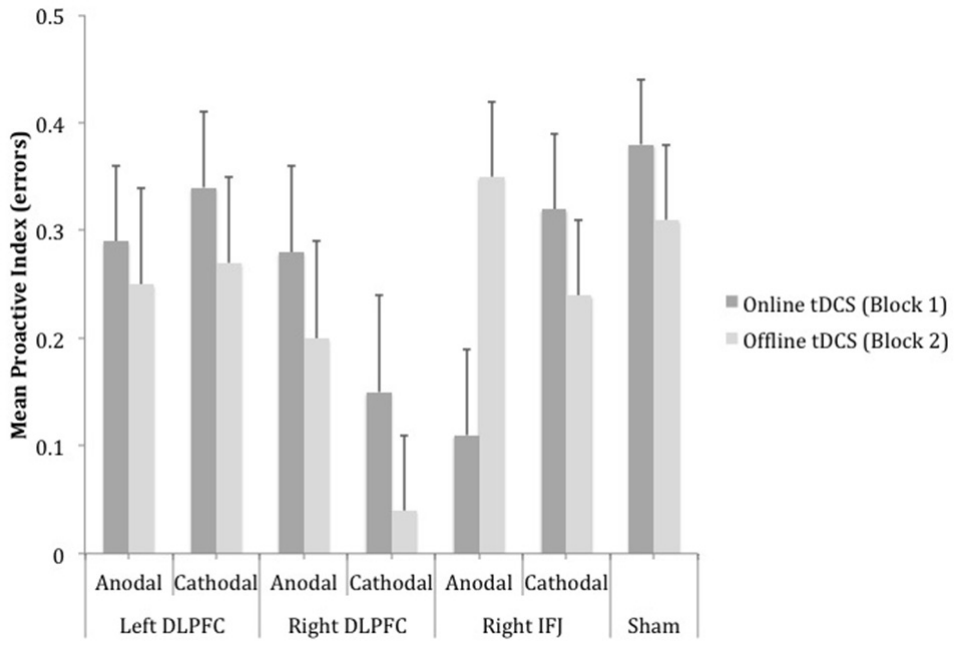
**Mean scores of the error-based proactive index as a function of site, polarity and timing of stimulation**. Bars represent standard error of mean (DLPFC, Dorsolateral Prefrontal Cortex; IFJ, Inferior Frontal Junction).

A mixed ANOVA (group x block) on the proactive index based on errors failed to show a reliable effect of group [*F*_(6, 157)_ = 1.53, MSE = 0.02, *p* = 0.17, partial η^2^ = 0.06] and block [*F* < 1, partial η^2^ = 0.00] (see Figure 4). However, the interaction group × block became marginally significant, *F*_(6, 157)_ = 1.91, MSE = 0.10, *p* = 0.08, partial η^2^ = 0.07, which appeared to be accounted for by changes in performance in cathodal right DLPFC and anodal right IFJ relative to sham. In support of this, the 3 (cathodal right DLPFC, anodal right IFJ, and sham) × 2 (online and offline) interaction was reliable and had a relatively large effect size, [*F*_(2, 67)_ = 5.20, MSE = 0.09, *p* < 0.01, partial η^2^ = 0.13], whereas the interaction involving the remaining stimulation groups and sham was not, [*F*_(4, 112)_ < 1, MSE = 0.09, partial η^2^ = 0.00]. Simple effect analyses to follow up the reliable interaction showed that there were differences between the groups at both blocks [online: *F*_(2, 67)_ = 3.35, MSE = 0.15, *p* < 0.05, partial η^2^ = 0.09; offline: *F*_(2, 67)_ = 5.22, MSE = 0.13, *p* < 0.01, partial η^2^ = 0.13]. *Post-hoc* tests within each block revealed that the only statistically significant difference in the online block concerned anodal right IFJ, which exhibited lower proactive indices than sham (*p* = 0.04; the difference between cathodal right DLPFC and sham did not reach statistical significance: *p* = 0.11). In the offline block, however, it was cathodal right DLPFC that showed a statistically significant decrease in the proactive index relative to anodal right IFJ (*p* = 0.01) and sham (*p* = 0.03), which did not differ from each other (*p* = 0.91).

**Figure 5:**
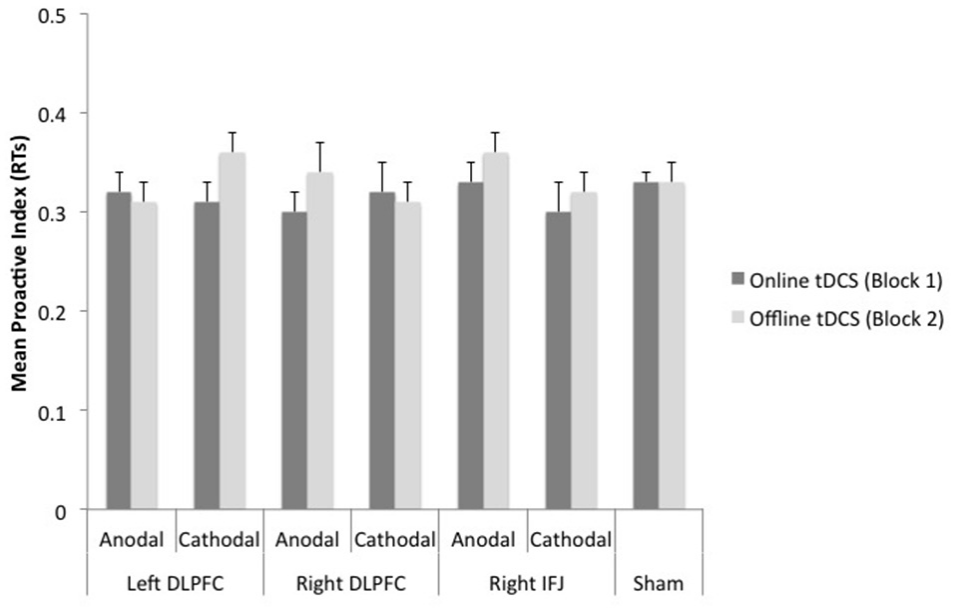
**Mean scores of the reaction time-based proactive index as a function of site, polarity, and timing of stimulation**. Bars represent standard error of mean (DLPFC, Dorsolateral Prefrontal Cortex; IFJ, Inferior Frontal Junction).

The ANOVA 7 (group) × 2 (block) on the proactive index based on RTs showed a marginally significant effect of block, *F*_(1, 150)_ = 3.31, MSE = 0.007, *p* = 0.07, partial η^2^ = 0.02 (see Figure 5). The proactive index tended to be lower while tDCS was being delivered (*M* = 0.33) than after it (*M* = 0.34). Neither group [*F*_(6, 150)_ < 1, partial η^2^ = 0.02] nor the interaction group x block [*F*_(6, 150)_ = 1.03, MSE = 0.006, *p* = 0.41, partial η^2^ = 0.03] reached statistical significance.

## Discussion

The results of the present study clearly indicate that tDCS may modulate performance on the AX-CPT, with such an effect depending on stimulation site, current polarity, and timing of stimulation. In this respect, it is important to note that the effect of tDCS on performance was far from generalized, since it essentially altered the pattern of errors for conflict trials. A number of findings merit discussion and are addressed below.

Paralleling the pattern observed in other studies with young participants that used similar versions of the AX-CPT (e.g., Morales et al., 2013), the sham group exhibited a straightforward strategy of proactive control, as reflected by the high scores in the proactive indices stemming from a worse performance in AY than in BX trials. Importantly, this pattern was the same for the two blocks of trials, which enables us to use this group's performance to assess the potential behavioral effects of tDCS in the stimulation groups.

Regarding real stimulation, tDCS over the left DLPFC failed to modulate performance in AY and BX trials. Neither anodal nor cathodal stimulation led to changes in the proactive indices as compared to sham stimulation in any of the two blocks of the task. This finding is somehow surprising because neuroimaging studies have shown that activity in the left DLPFC correlates with performance changes in the AX-CPT (e.g., Braver et al., 2009; Lesh et al., 2013; see also Lopez-Garcia et al., 2016). In addition, a vast experimental literature endorses the role of this region in tasks that involve information maintenance and manipulation (e.g., Barbey et al., 2013; Tremblay et al., 2014). There was, however, an effect of anodal tDCS of the left DLPFC on responses to target trials. Specifically, participants from this group, in comparison to those from the sham group, responded faster in the AX condition during the offline block, which points to a stimulation-induced benefit that was specifically limited to the “yes” (most frequent) trials. Some studies have reported stimulation-related changes in working memory functioning (in particular enhanced performance associated with anodal tDCS: Ohn et al., 2008; Andrews et al., 2011; Hussey et al., 2015), even though these effects tend to be small, and other studies have failed to observe such changes (for meta-analytic reviews, see Brunoni and Vanderhasselt, 2014; Hill et al., 2016; Mancuso et al., 2016). All in all, a straightforward prediction in the present study was that tDCS of the left DLPFC would modulate performance in AY and BX trials relative to sham. It should be noted that while clearly demanding active maintenance of task-relevant information, the AX-CPT might be better described as a conflict resolution task with only moderate memory load requirements. Hence, the putative tDCS-induced modulation of activity in the left DLPFC could not be enough to allow for the observation of changes in cognitive control. The fact that neither anodal nor cathodal tDCS led to shifts in the proactive indices in any of the two trial blocks points to this possibility. The involvement of the left DLPFC in shifting between cognitive controls strategies should be further explored in future tDCS studies by using stimulation protocols other than the one used in the present study.

A very different pattern of performance emerged when tDCS was delivered over the right hemisphere. Supporting its involvement in the balance between proactive and reactive control (Braver et al., 2009), the application of anodal tDCS over the right IFJ led participants to adopt a more reactive strategy (as reflected by lower scores in the proactive index based on errors) when responding to the first block of trials. Importantly, this effect did not persist over the second block, which indicates that anodal tDCS induced a behavioral change that specifically affected the conflict trials (making BX errors increase relative to AY errors), but only when the electrical current was being delivered; performance throughout the block with tDCS offline was comparable to that observed in the sham group, where participants exhibited the proactive pattern typically observed in young healthy people. Anodal tDCS of the right IFJ also made participants respond faster to target (AX) trials in the second than in the first block.

Our expectation that tDCS of the right IFJ would modulate performance on the AX-CPT was based on neuroimaging data showing that this region exhibits bidirectional shifts in activation dynamics that are associated with shifts in behavioral measures (Locke and Braver, 2008; Paxton et al., 2008; Braver et al., 2009; for related results using a different task, see Han and Marois, 2014). According to Braver et al. (2009), this bidirectional dynamic is suggestive of the highly flexible activation response of certain regions of the lateral PFC, which might reflect individual differences in which cognitive control mode is deployed by default (e.g., younger people vs. older people), as well as control mode shifts occurring within individuals as a consequence of changing task conditions. Our finding regarding the right IFJ supports this idea. Specifically, the increase observed in the BX error rate (relative to AY errors) after (anodal) tDCS of this lateral prefrontal region points to its involvement in shifting the focus of processing for relevant information. Following the usual interpretation of the proactive indices from the AX-CPT (e.g., Braver et al., 2009), the anodal right IFJ group's performance is indicative of higher reliance on probe processing as compared to that of the sham group, since this control mode is expected to bring increased errors in BX trials since the probe signals a “yes” response (in the context of a task where 70% of the trials with an X probe elicit a “yes”).

While its precise function in cognitive control is far from clear, a good deal of research points to the pivotal role that the IFJ plays in orchestrating thoughts and actions in accordance with internal goals (Bunge et al., 2003; Brass and von Cramon, 2004; Derrfuss et al., 2004, 2005; Brass et al., 2005; Verbruggen et al., 2010; Sundermann and Pfleiderer, 2012; Han and Marois, 2014). Thus, and because of its connections with other cortical regions (see, for example, Sundermann and Pfleiderer, 2012), some authors have posited that the functional role of the IFJ may be related to the activation of task representations to allow for flexible adjustment to a changing environment (Brass et al., 2005). It has also been suggested that the IFJ, as part of a cognitive control network that includes other frontal regions operating in a more specific way, may be generally involved in task switching and flexibility (Kim et al., 2012; Sundermann and Pfleiderer, 2012). In this respect, recent functional connectivity studies have attributed to the IFJ and adjacent areas the role of an integration hub responsible for coordinating the processing flow while performing complex cognitive tasks (Cole et al., 2013; Sneve et al., 2013; see also Han and Marois, 2014). Additionally, a recent study that used a form of repetitive transcranial magnetic stimulation to disrupt neural function in the right IFJ concluded that this area seems to be specifically involved in detecting infrequent but relevant task-relevant stimuli in order to update behavior in a changing environment (Verbruggen et al., 2010; see also the review by Levy and Wagner, 2011), for a related interpretation of the role of the right IFJ in cognitive control). Within this framework, our results could be indicating that anodal tDCS of the right IFJ disrupted its normal activity, which would be more evident in the (conflict) trials with the highest demands of coordination in accordance with the task goals. Specifically, because an X probe is strongly associated with a prepotent target response (given the high prevalence of AX trials), BX trials represent the considerable challenge of avoiding giving a “yes” response. Hence, tDCS could have hindered the detection and/or features processing of infrequent (20% of trials) non-A cues, especially in the presence of multiple distractors as was shown to be the case in the AX-CPT used here, which would prompt high reliance on probe processing. This stimulation-induced disruption of cognitive control during the first block contrasts with the facilitation effect observed for RT during the second block, which exclusively concerned target (no conflict) trials.

Stimulation also led participants to respond by conforming to a more reactive mode of cognitive control when applied over the right DLPFC which, unlike the right IFJ, was modulated by cathodal stimulation but only during the second (offline tDCS) block (performance during the first block seemed to also be impacted by cathodal stimulation when compared with sham, but the difference did not survive *post-hoc* tests). Our observation that stimulating the right DLPFC modulates performance on the AX-CPT is not surprising in light of a number of previous studies and theoretical proposals suggesting that this area plays a pivotal role as part of a distributed network in cognitive control (e.g., Smith and Jonides, 1999; Koechlin et al., 2003; Ridderinkhof et al., 2004; Tanji et al., 2007; Klein et al., 2007). However, as fMRI studies using the AX-CPT have led us to assume that the right DLPFC might have a lesser involvement than its contralateral counterpart or the right IFJ in shifting the mode of cognitive control (Braver et al., 2009), we expected tDCS of the right DLPFC to be less effective at inducing changes in performance than stimulation of the other two prefrontal regions. In any case, the fact that tDCS of the right DLPFC reliably induced a change in the manner the participants dealt with conflict trials supports the involvement of this area in executive control.

The right DLPFC, along with other prefrontal and subcortical regions, is typically considered a part of an inhibitory network (Kelly et al., 2004; Beeli et al., 2008; Shackman et al., 2009; Gagnepain et al., 2014; Cipolotti et al., 2016; but see Aron et al., 2014). Thus, for example, this region is thought to play a role in the distributed neural system underpinning behavioral inhibition in response to threat, with more behaviorally inhibited individuals showing more EEG activity in the right DLPFC (McNaughton and Corr, 2004; Shackman et al., 2009). Activity in this right-lateralized prefrontal area has also been shown to predict decreased activity in the hippocampus and successful intentional episodic forgetting, which has been interpreted in terms of memory inhibition (Anderson and Hanslmayr, 2014). Along these lines, a recent study showed that cathodal tDCS over the right DLPFC specifically made participants unable to intentionally forget, which suggests that stimulation disrupted the normal activity of said region to downregulate memory accessibility (Silas and Brandt, 2016). It remains unclear, however, which precise role the right DLPFC plays in the inhibitory network. Thus, some authors have related its activity to response selection processes that flexibly allow for adaptation to changing environments (e.g., Rowe et al., 2000; Bunge et al., 2002; Jiang and Kanwisher, 2003). From this perspective, and because successfully responding to conflict trials in the AX-CPT necessarily demands response selection, it seems reasonable to suggest that in the present study cathodal tDCS of the right DLPFC hindered performance by disrupting probe-related response selection, which led participants to make more errors in BX trials (increased error rates in BX trials relative to AY in comparison with sham; it should be noted that a general impairment in selecting the appropriate response would have also led to more errors in AY trials, where the cues but not the probes signal a “yes” response). Alternatively, it has been suggested that the right DLPFC is involved in preventing stimulus-induced activation of irrelevant but prepotent stimulus-response bindings (Kühn et al., 2011; Zmigrod et al., 2014). By using an experimental task that requires participants to create stimulus-response bindings, Zmigrod et al. (2014) showed that tDCS of the right, but not the left, DLPFC made young healthy participants' performance comparable to that of populations thought to suffer from executive control impairments derived from prefrontal dysfunction (e.g., Hommel et al., 2011). This finding joins previous results from a variety of brain-related studies (e.g., Kelly et al., 2004) in pointing to the role that the right DLPFC may play in downregulating stimulus-induced activation of irrelevant event representations. From this perspective, the increased rate of “yes responses” to BX trials in our cathode right DLPFC group could be interpreted as a consequence of an impairment in modulating the influence of dominant, integrated stimulus-response representations (“X-yes response”). It is worth noting here that while the two above-mentioned interpretations attribute distinct roles to the right DLPFC, both fit well with the general assumption that this region is a core component of the inhibitory control network (i.e., Kelly et al., 2004; Shackman et al., 2009; Gagnepain et al., 2014; Zmigrod et al., 2014; Cipolotti et al., 2016), which could have been compromised by cathodal tDCS in the present experiment.

A remarkable finding of the present study is the dissociation of the behavioral effect of tDCS over two right-lateralized prefrontal regions as a function of polarity and stimulation timing. Thus, cathodal tDCS of the DLPFC and anodal tDCS of the IFJ had a similar impact on cognitive control, even though with a different timing. On one hand, this finding strongly points to the relevance of considering the existence of state-dependent effects when it comes to investigating the behavioral outcomes of brain stimulation. Although we are blind here regarding the possible aftereffects of only stimulating the right DLPFC (without concurrently performing the AX-CPT), the results of a number of studies that used different stimulation techniques reveal that performing a cognitive task induces neural states that interact with the application of stimulation (Silvanto et al., 2008; Romei et al., 2016), and that the effects of tDCS may be different as a function of ongoing cognitive/motor activity (Fritsch et al., 2010; Fertonani et al., 2014).

Different neurophysiological mechanisms could have mediated the comparable behavioral outcomes observed for cathodal right DLPFC and anodal right IFJ. Online tDCS effects are thought to mainly stem from membrane polarization (stimulation would modulate the likelihood that neurons will fire by hyperpolarizing or depolarizing the brain tissue; but see Romero Lauro et al., 2014 for changes in cortical excitability 15 min after stimulation completion), whereas tDCS offline (prolonged) effects have been attributed to post-synaptic modifications mediated by long-term potentiation/depression-like mechanisms (Nitsche et al., 2003; Stagg et al., 2009) as well as to neurons' membrane alterations (Ardolino et al., 2005). Without recording and monitoring tDCS-related brain activity, however, we can only speculate about this possibility and highlight the relevance of addressing this issue in future studies. In this respect, and as suggested above, the observed effect of cathodal tDCS of the right DLPFC could be reliant on the active state of the stimulated area (or network), since participants were being stimulated while performing the first block of the AX-CPT. This is another issue that warrants further investigation to improve our understanding of the neural substrate of cognitive control and flexibility.

On the other hand, the observed dissociation is suggestive of the relative site-specific nature of the effect of our stimulation protocol (using an extracephalic return electrode; see Woods et al., 2016), since both areas are relatively close to one another (F4 and midway FC4-FC6). If it were otherwise, the behavioral change relative to sham would have been observed under the same tDCS conditions for the right DLPFC and the IFJ groups. In addition, it is worth mentioning that the fact that anode and cathode (over different timings and locations) gave rise to similar performances goes against the now well-established, oversimplified idea that increased neural excitability produced by anodal tDCS leads to enhanced performance, whereas worsened performance follows decreased neural excitability produced by cathodal tDCS (Jacobson et al., 2012; Bestmann et al., 2015; Fertonani and Miniussi, 2016; Woods et al., 2016). In this sense, our results join others to reveal that tDCS of the lateral right prefrontal cortex, regardless of polarity, may hinder performance (e.g., Zmigrod et al., 2014). As a matter of fact, stimulating the right DLPFC or the ipsilateral IFJ made healthy young adults' behavior comparable to that observed in older adults or individuals with altered PFC functions, who usually exhibit impoverished proactive cognitive control (e.g., Braver et al., 2001; Barch et al., 2001; Holmes et al., 2005; Paxton et al., 2008). This strongly supports the relevance of tDCS to create temporary brain dysfunctions that allows for a more casual approach to investigating the neural correlates of behavior (Filmer et al., 2014; Bestmann et al., 2015).

As previously mentioned, facilitated performance during the second block was observed after anodal tDCS of either the left DLPFC or the right IFJ, even though it modestly affected response times to target trials. Thus, this effect emerged in conditions wherein tDCS failed to modulate cognitive control. Since the different role that both prefrontal regions may play in successfully performing the AX-CPT, the absence of stimulation-related predictions concerning AX trials, and the noticeably restricted nature of the observed enhancement, it is not obvious to us the cognitive mechanism/s underlying the increase in response speed in the second block. Despite this, the observation of faster responses after anodal stimulation does not come as too much of a surprise (see, for example, Tremblay et al., 2014). Anodal tDCS could have amplified (e.g., through mechanisms related to long-term potentiation; Stagg et al., 2009) the effect of practice for target trials in the context of a relatively long and somewhat dull task, wherein fatigue and diminished alertness during the second block would be expected to counteract the benefit of practice during the first block. Again, this finding supports the idea that the behavioral outcomes of tDCS may depend on certain neural states induced by the task at hand (Fertonani et al., 2014; Romei et al., 2016).

To conclude, the present findings join others from previous fMRI studies pointing to the involvement of the right DLPFC and the right IFJ in shifting between distinct modes of cognitive control, which is thought to reflect the flexible nature of human cognition. Furthermore, in what can be seen as a novel contribution to the field, the present study provides the very first piece of causal evidence of such an involvement, since neural activity in these target (and presumably connected) areas was directly altered by tDCS, which in turn hindered performance. While we also found that stimulation did not have a behavioral effect when delivered over the left DLPFC, another prefrontal area that has been systematically associated with cognitive control, our results as a whole suggest that tDCS might be used to induce changes in the way cognitive control is deployed, at least in healthy young adults.

## Author contributions

CG is the principal responsible for the conception and design of the study and wrote the manuscript. MM contributed to the design of the study, data acquisition and analyses, and manuscript editing. JM contributed to the theoretical background, preparation of the experimental task and design, data analyses, and manuscript editing.

## Funding

The current study was completed thanks to financial support by grants from the Spanish Ministerio de Economía y Competitividad to CG (PSI2011-25797 and PSI2015-65502-C2-2-P).

### Conflict of interest statement

The authors declare that the research was conducted in the absence of any commercial or financial relationships that could be construed as a potential conflict of interest.
